# A Follow-up Study on the Trajectory and Sex Differences of Different Cognitive Dimensions in the Normal Aging Elderly in Guangzhou

**DOI:** 10.31083/AP44186

**Published:** 2025-06-24

**Authors:** Jie Dong, Chan Su, Ruoxi Zhang, Xiong Luo, Ruoyan Huang, Bin Sun, Jia Li, Muni Tang

**Affiliations:** ^1^Department of Psychiatry, Hainan General Hospital/Hainan Affiliated Hospital of Hainan Medical University, 570100 Haikou, Hainan, China; ^2^Department of Geriatric Psychiatry, The Affiliated Brain Hospital, Guangzhou Medical University, 510145 Guangzhou, Guangdong, China

**Keywords:** healthy aging, sex characteristics, cognition

## Abstract

**Objective:**

To explore trajectory and sex differences of varied cognitive dimensions over time in the normal aging elderly.

**Methods:**

The cluster sampling method was used to select a representative community (n = 341). Individuals aged 60 years and above with normal cognitive function were identified (n = 281). One-to-one neuropsychological tests were conducted at baseline and repeated 1 and 5 years later. A mixed linear model was developed to analyze the changes and sex discrepancies in different cognitive dimensions of the elderly based on the scores of the Mini-Mental State Examination (MMSE), Montreal Cognitive Assessment (MoCA), and World Health Organization Battery of Cognitive Assessment Instruments for the elderly (WHO-BCAI).

**Results:**

When comparing the 1-year follow-up with baseline data, the scores of auditory vocabulary in both men and women had significantly increased (*t* = –2.52, *t* = –4.8, *p* < 0.05), while the Wechsler mapping in women had significantly increased (*t* = –2.62, *p* < 0.05). When comparing the 5-year follow-up with baseline data, MMSE (*t* = 6.38, *t* = 6.06, *p* < 0.05) and MoCA (*t* = 7.34, *t* = 7.03, *p* < 0.05) scores had significantly decreased in both groups, the digit span scores had significantly decreased in men (*t* = 2.67, *p* < 0.05), and the scores of auditory vocabulary in women had significantly increased (*t* = –2.54, *p* < 0.05). When comparing the 5-year follow-up with the 1-year follow-up data, the digit span scores in women had significantly decreased (*t* = 2.88, *p* < 0.05), and the Wechsler mapping scores in both groups had significantly decreased (*t* = 3.68, *t* = 3.14, *p* < 0.05). A sex discrepancy emerged in several tests of specific cognitive domains after adjusting for education. At baseline, women outperformed men in auditory vocabulary, associative learning, and delayed recall while men outperformed women in Wechsler mapping. Importantly, auditory vocabulary and Wechsler mapping were better in women and men, respectively, at all visit times. The superiority of associative learning and delayed recall of women shrunk with advancing age.

**Conclusions:**

Sex differences in cognitive variation indicated a steeper decline for women in verbal episodic memory, associative memory, and short-term memory. Meanwhile, a sharper deterioration in memory, comprehension and recognition of spatial relationships, and visual structure ability was observed in men. The sex differences in different cognitive dimensions diminished over time.

## Main Points

1. This study reveals the trajectory of different cognitive dimensions in men and women respectively in normal aging.

2. This study showed that verbal episodic memory, associative memory, and short-term memory decrease more rapidly in women, while visual structure, recognition of spatial relationships, memory, and comprehension declined more sharply in men.

3. The discrepancy of the cognitive performance in men and women gets lessened over time.

## 1. Introduction

With the aging population is getting unprecedented huge, it has become a universal topic worldwide of healthy aging [1]. It is estimated that the global population of individuals aged 65 and above will reach to 1.5 billion and life expectancy will on average increase to 77 years by 2050 [2]. Indeed, cognitive health is the promise of healthy aging, and taking sex into account of what is normal in cognitive aging and how to develop differential strategies to alleviate impairments to cognitive health is very important. Cognition refers to a process of knowing and reasoning, in which memory is the key process to store and retain information [3]. Cognitive ability involves a variety of dimensions, such as logic reasoning, processing speed, attention, memory, language, executive function, visuospatial ability and so on [4]. Our longitudinal study has found that when analyzed according to age, the continuous attention, short-term memory, and recognition, memory and comprehension of visuospatial structures in the younger age group declined faster than older age groups [5]. Small studies have focused on the feature of normal aging process in different sex individuals, yet the conclusions have long been controversial [6, 7]. Some studies found that women’s episodic memory is generally better than that of men, whereas men’s information-processing abilities are better than women [8, 9, 10]. However, some studies have found no differences [11, 12]. It tends to be consistent that men perform better than women in mental rotation and visuospatial tasks, whereas women perform better than men in verbal and positional memory [13, 14]. Nevertheless, small-to-nonexistent sex or sex differences was found on geometry in mathematics tests [15]. It was also reported that women excel men in faces, objects and numbers’ memory tasks [16]. Not surprisingly, there were findings disconfirmed the conclusion [17]. Based on the debates of cognitive sex differences, we proposed the assumption that old men and women hold their advantage in specific cognitive dimension throughout normal aging, respectively. Yet the advantage orientation as well as its extent will change over time. This study aimed to explore the trajectory of varied cognitive dimensions (including attention, memory, visual spatial and executive function) of the normal cognitive and different sex elderly across aging, which was expected to shed a light on the road to prevention and early intervention of sex-specific cognitive impairment in the elderly.

## 2. Methods

### 2.1 Participants 

A total of 341 subjects were selected from an urban area of Guangzhou by cluster sampling method and 281 subjects were finally enrolled. Inclusion criteria: (a) individuals aged 60 years and above; (b) the resident population of the surveyed area and the non-resident population living in the survey area for more than 1 month; (c) agreed to accept the survey and signed the informed consent. Exclusion criteria: (a) permanent residents who have been away for more than 1 month; (b) non-residents who have lived in the survey place for less than 1 month; (c) individuals suffering from severe functional mental illnesses, physical diseases, deafness, visual impairment, or inability to complete the examination. Loss of follow-up criteria: (a) failed to be found three times at different time; (b) refuse to be persuaded for three times at different time. This study has been reviewed by the Ethics Committee meeting of Shanghai Mental Health Center, and the ethics review number is 2012-19. All subjects or their guardians in this study had been given informed consent to this study and signed informed consent forms.

### 2.2 Procedures

Baseline: 2011, the first follow-up: 2012, the second follow-up: 2016. Assessment tools: (1) Overall cognitive function assessment: Mini-mental State Examination (MMSE): compiled by Folstein in 1975, translated and revised by Cai *et al*. [18]. Montreal Cognitive Assessment (MoCA): Developed by Nasreddine *et al*. [19], Canada. The Beijing version was used in this Putonghua version. (2) Assessment of different dimensions of cognitive function: World Health Organization-battery of cognitive assessment instrument for elderly, World Health Organization-battery of cognitive assessment instrument for elderly (WHO-BCAI). It was developed by the World Health Organization for the elderly, with moderate difficulty, and is suitable for the elderly from different countries and cultural backgrounds. The Department of Geriatrics of the Mental Health Center Affiliated to Shanghai Jiao Tong University introduced and edited the formulation of the Chinese norm, which has good reliability and validity [20].

Investigators: The questionnaire investigators of the baseline, first follow-up (1 year later) and second follow-up (5 years later) were all junior undergraduates majoring in psychology from a same university. Unified investigation procedures and standards were stipulated. The same teacher was invited to provide systematic training for the above-mentioned investigators for 2 weeks in all three surveys, and individual investigation test was conducted for each investigator after training. Only qualified investigators were granted the qualification. Each time all investigators have a conformance test, and the survey can be conducted only when the Kappa value ≥0.8.

Data processing and analysis: EpiData3.0 software (the EpiData Association, Odense, Denmark) was used to establish a database, data were entered twice, and cleaning error detection and logical analysis were carried out by comparison; SPSS26.0 (IBM Corp., Armonk, NY, USA) was used to analyze the data, and descriptive statistics, variance analysis, mixed linear model and other methods were used to analyze the data. A histogram was used for normality testing and the Bonferroni method for post hoc testing. *p* < 0.05 was considered to be statistically significant.

Related definitions: the old with normal cognitive function: ① aged 60 years and above; ② with normal cognitive function; ③ do not meet the diagnostic criteria for mild cognitive impairment proposed by Petersen *et al*. [21] and the Diagnostic and Statistical Manual of Mental Disorders, Fourth Edition (DSM-IV) of the American Psychiatric Association for various types of dementia.

## 3. Results

### 3.1 Basic Demographic Data

Baseline: A total of 341 individuals were examined, of whom 281 were elderly individuals with normal cognitive function (139 men and 142 women; average age, 70.08 ± 7.97 years).

At the first follow-up, 282 of 341 individuals were checked, of whom 218 were elderly individuals with normal cognitive function (103 men and 115 women; average age, 69.92 ± 7.65 years).

In the second follow-up, 210 of 282 individuals were checked, including 162 elderly people with normal cognitive function (77 men and 85 women; average age, 74.12 ± 7.68 years).

### 3.2 Factors Influencing Cognitive Function

Multivariate Analysis of Variance (ANOVA) was performed with cognitive test scores defined as dependent variables and sex, age, and education level as independent variables, and the results showed that sex had no significant effect on cognitive function, while age and education had an impact on cognitive function. The intervariate test showed that age had a significant effect on MMSE, MoCA, auditory vocabulary, associative learning test, verbal fluency test, delayed recall, Wechsler mapping and Wechsler block, but had no effect on the digit span test. Education had a significant effect on the results of MMSE, MoCA, Wechsler mapping and Wechsler Block, but had no significant effect on the other cognitive dimensions (Table 1).

**Table 1.** **Factors influencing cognitive function**.

Item	Sex		Age		Education		Sex*Education		Sex*Age		Age*Education		Sex*Age*Education	
	*F*	*p*	*F*	*p*	*F*	*p*	*F*	*p*	*F*	*p*	*F*	*p*	*F*	*p*
Overall	1.23	0.28	2.03	0.01	1.48	0.02	0.85	0.74	0.55	0.93	0.91	0.69	1.14	0.24
Variable														
MMSE	0.16	0.69	3.78	0.03	4.61	0.00	0.57	0.73	0.03	0.97	0.47	0.89	0.33	0.89
MoCA	1.04	0.31	8.86	0.00	4.95	0.00	1.35	0.25	1.15	0.32	1.51	0.15	1.48	0.20
Digit span	0.05	0.82	0.29	0.75	0.14	0.98	0.08	1.00	0.01	0.99	0.24	0.99	0.05	1.00
Auditory vocabulary	0.42	0.52	5.21	0.01	1.00	0.42	1.54	0.18	0.39	0.68	0.92	0.51	2.43	0.04
Associative learning	1.25	0.27	4.90	0.01	0.83	0.53	0.55	0.74	0.89	0.41	1.38	0.20	1.61	0.16
Verbal fluency	0.31	0.58	4.58	0.01	1.84	0.11	0.78	0.57	2.19	0.12	1.08	0.41	1.84	0.11
Delayed recall	3.25	0.07	6.69	0.00	1.35	0.25	0.87	0.50	1.25	0.29	0.91	0.52	1.48	0.20
Wechsler mapping	3.27	0.07	6.24	0.00	3.82	0.00	0.37	0.87	0.60	0.55	1.58	0.13	0.89	0.49
Wechsler block	0.78	0.38	5.50	0.01	2.62	0.03	0.85	0.52	0.62	0.54	0.72	0.69	0.83	0.53

MMSE, Mini-mental State Examination; MoCA, Montreal Cognitive Assessment. * means the interaction effect of the two factors.

### 3.3 Cognitive Function Change of Sex Discrepancy Over Time

The scores of auditory vocabulary, associative learning, delayed recall, and Wechsler mapping among the elderly with normal cognitive function of different sexes were statistically significant at baseline. The interaction effects of sex and time was not significant in all cognitive test dimensions (education has been adjusted) (Table 2).

**Table 2.** **Comparison of different cognitive dimensions among male and female elderly with normal baseline cognition at various survey periods $(\bar{x}\pm s$)**.

	Overall			Men			Women			Sex		Time		Sex * Time	
	B	V1	V2	B	V1	V2	B	V1	V2	*F*	*p*	*F*	*p*	*F*	*p*
	n = 281	n = 218	n = 162	n = 139	n = 103	n = 77	n = 142	n = 115	n = 85						
MMSE	25.85 ± 0.20	25.42 ± 0.22	23.64 ± 0.25	25.74 ± 0.29	25.52 ± 0.32	23.38 ± 0.37	25.96 ± 0.29	25.32 ± 0.31	23.91 ± 0.34	0.26	0.61	40.73	0.00	1.06	0.35
MoCA	20.94 ± 0.25	21.01 ± 0.27	18.10 ± 0.30	21.22 ± 0.37	21.30 ± 0.39	18.21 ± 0.45	20.66 ± 0.36	20.72 ± 0.38	17.99 ± 0.42	0.93	0.34	64.03	0.00	0.23	0.80
Digit span	7.60 ± 0.27	7.69 ± 0.28	7.04 ± 0.30	7.65 ± 0.38	7.52 ± 0.39	6.93 ± 0.42	7.55 ± 0.38	7.86 ± 0.39	7.14 ± 0.40	0.09	0.77	6.52	0.00	1.16	0.32
Auditory vocabulary	6.37 ± 0.13	7.12 ± 0.14	6.87 ± 0.17	6.02 ± 0.19	6.55 ± 0.21	6.41 ± 0.25	6.72 ± 0.19	7.69 ± 0.20	7.33 ± 0.23	15.75	0.00	13.53	0.00	1.08	0.34
Associative learning	5.40 ± 0.20	5.61 ± 0.23	5.41 ± 0.28	4.89 ± 0.30	5.18 ± 0.32	5.15 ± 0.42	5.91 ± 0.29	6.03 ± 0.32	5.66 ± 0.37	4.95	0.03	0.39	0.68	0.36	0.70
Verbal fluency	7.96 ± 0.17	8.41 ± 0.19	8.30 ± 0.23	7.78 ± 0.24	8.44 ± 0.27	8.08 ± 0.34	8.14 ± 0.24	8.38 ± 0.26	8.52 ± 0.31	0.75	0.39	2.55	0.08	0.69	0.51
Delayed recall	4.97 ± 0.17	5.11 ± 0.19	4.82 ± 0.22	4.36 ± 0.25	4.29 ± 0.27	4.41 ± 0.32	5.58 ± 0.24	5.93 ± 0.26	5.24 ± 0.30	16.65	0.00	0.79	0.45	1.62	0.20
Wechsler mapping	9.29 ± 0.20	9.92 ± 0.21	8.68 ± 0.25	10.17 ± 0.28	10.68 ± 0.30	9.28 ± 0.37	8.41 ± 0.28	9.16 ± 0.30	8.07 ± 0.35	17.78	0.00	12.35	0.00	0.63	0.53
Wechsler block	24.64 ± 0.47	24.63 ± 0.50	23.69 ± 0.59	25.59 ± 0.67	25.61 ± 0.72	24.10 ± 0.87	23.69 ± 0.67	23.65 ± 0.71	23.27 ± 0.81	3.25	0.07	1.67	0.19	0.56	0.57

B, baseline (baseline); V1, Visit 1 (the first follow-up); V2, Visit 2 (the second follow-up). * means the interaction effect of the two factors.

### 3.4 Comparison of the Scores of Different Cognitive Dimensions in Different Survey Periods (Intragroup Differences)

Compared with the baseline and the first follow-up, the second follow-up MMSE and MoCA scores decreased significantly in both men and women. Compared with the baseline, the second follow-up scores of the digit span of men decreased significantly, whereas compared with the first follow-up, the second follow-up scores of the digit span of women decreased significantly. As for auditory vocabulary, the first follow-up increased significantly in both men and women compared to baseline, and the second follow-up showed a significant increase only in women. Scores of associative learning, verbal fluency, delayed recall, and the Wechsler block showed no statistically differences between men and women in multiple comparisons of baseline, first follow-up, and second follow-up. The Wechsler mapping scores of women at the first follow-up increased significantly compared with the baseline. Both men and women’s scores decreased significantly at the second follow-up compared to the first follow-up. Further details are presented in Table 3.

**Table 3.** **Comparison of cognitive test results at different survey times (intragroup differences) (score, $\bar{x}\pm s$)**.

Test	Women									Men								
	V1/B			V2/B			V2/V1			V1/B			V2/B			V2/V1		
	Difference in means	*t*	*p*	Difference in means	*t*	*p*	Difference in means	*t*	*p*	Difference in means	*t*	*p*	Difference in means	*t*	*p*	Difference in means	*t*	*p*
MMSE	0.64 ± 0.31	2.06	0.11	2.06 ± 0.34	6.06	0.00	1.42 ± 0.35	4.06	0.00	0.22 ± 0.31	0.71	1.00	2.36 ± 0.37	6.38	0.00	2.14 ± 0.38	5.63	0.00
MoCA	0.06 ± 0.34	0.18	1.00	2.67 ± 0.38	7.03	0.00	2.74 ± 0.39	7.03	0.00	0.08 ± 0.34	0.24	1.00	3.01 ± 0.41	7.34	0.00	3.09 ± 0.42	7.36	0.00
Digit span	0.31 ± 0.21	1.48	0.43	0.40 ± 0.24	1.67	0.30	0.72 ± 0.25	2.88	0.01	0.14 ± 0.21	0.67	1.00	0.72 ± 0.27	2.67	0.03	0.59 ± 0.28	2.11	0.11
Auditory vocabulary	0.96 ± 0.20	–4.8	0.00	0.61 ± 0.24	–2.54	0.03	0.36 ± 0.25	1.44	0.45	0.53 ± 0.21	–2.52	0.04	0.39 ± 0.25	1.56	0.34	0.14 ± 0.26	0.54	1.00
Associative learning	0.12 ± 0.35	0.34	1.00	0.24 ± 0.40	0.06	1.00	0.37 ± 0.42	0.88	1.00	0.29 ± 0.36	0.81	1.00	0.26 ± 0.44	0.59	1.00	0.03 ± 0.46	0.07	1.00
Verbal fluency	0.24 ± 0.29	0.83	1.00	0.39 ± 0.33	1.18	0.74	0.14 ± 0.34	0.41	1.00	0.66 ± 0.30	2.2	0.08	0.30 ± 0.36	0.83	1.00	0.36 ± 0.38	0.95	1.00
Delayed recall	0.35 ± 0.27	1.30	0.57	0.35 ± 0.30	1.17	0.75	0.70 ± 0.31	2.26	0.08	0.08 ± 0.28	0.29	1.00	0.05 ± 0.32	0.16	1.00	0.12 ± 0.34	0.35	1.00
Wechsler mapping	0.76 ± 0.29	–2.62	0.03	0.34 ± 0.34	1.00	0.97	1.10 ± 0.35	3.14	0.01	0.5 ± 0.29	1.72	0.24	0.88 ± 0.36	2.44	0.05	1.40 ± 0.38	3.68	0.00
Wechsler block	0.05 ± 0.65	0.08	1.00	0.42 ± 0.77	0.55	1.00	0.37 ± 0.79	0.47	1.00	0.02 ± 0.66	0.03	1.00	1.49 ± 0.82	1.82	0.21	1.51 ± 0.84	1.80	0.23

## 4. Discussion

Our study found that in terms of auditory vocabulary the scores of women were always higher than that of men across the survey, indicating that elderly women have greater verbal learning and short memory abilities than men and tend to maintain the advantage over time, which is consistent with many scholars’ opinions that women were more advantaged on language abilities (verbal memory ability, vocabulary learning and expression) than men [17, 22]. Some noted that this may be related to estrogen and verified the protective effect of estrogen in cognitive aging in women [23]. On the other hand, estrogen is a potential factor contributing to worse performance in women and men on mental rotation test, which reflects the visuospatial function [13, 24, 25]. In the associative learning test at baseline, the scores of women were significantly higher than men, indicating that women’s associative learning ability is superior to men’s at a younger old age, which is consistent with the findings of previous study [26]. This may be related to the more capable verbal processing ability of women. Align with this is findings illustrating that men perform better than women in visuospatial tasks without verbal cues, whereas women can use their verbal advantages to memorize and narrow the gap with men when there are verbal cues [27]. Despite the superiority at baseline, there was no significant difference was found of associative learning in the first and second follow-ups, indicating that the advantage of women gradually lessened. That means the decline in verbal episodic memory and associative memory of women is steeper than men, which is in accordance with some former explorations [16, 28]. As to verbal fluency, women’s scores showed a significant upward trend, whereas men’s scores decreased significantly over time. This indicates that women are superior to men in terms of semantic representation and there is an increasing trend with the accumulation of life experience, similar to the findings of a previous study [29]. In Wechsler mapping, although the scores of men in the second follow-up were higher than that of women, the average scores of men declined significantly compared with baseline, suggesting that even if the spatial abstraction ability of men is better than women, the decline is greater as well, which is consistent with previous literatures [26, 30, 31]. In the Wechsler block, the scores of men were higher than women at baseline, whereas there was no significant difference between the two groups at the first and second follow-ups. This suggests that older men excel women in ability to recognize, remember, and understand spatial structures, but they no longer maintain this advantage with increasing age. Actually, several researchers have reported that men typically outperform women in visuospatial tasks, while women outperform men in verbal tasks [32]. From the perspective of evolutionary psychology, men must hunt for food in a natural environment, their visuospatial ability has been inherited as an advantage through natural selection [33]. Some scholars have found that men’s mental state, perception-motor speed, integration ability, and visual-spatial ability decline at a faster rate than women’s over time [34]. Studies have proposed that women are more likely to achieve successful aging than men, which may be related to the fact that elderly women have better social support systems because of their greater participation in family life [35, 36]. However, some studies have found that elderly women perform worse than men in terms of health, cognition, and other fields [37, 38]. As men have more opportunities to continue participating in social interactions and work, their high socioeconomic status is the main influencing factor [39]. It was also found that when women’s sex identity is fully valued and educational as well as employment opportunities are equally offered, their episodic memory performance will be better than men [40]. From a neuroanatomical point of view, regarding the white matter microstructure, women have lower directionality fractional anisotropy and higher tract complexity, even after adjusting for brain volume, which is related to women’s advantage in speech [41]. Our study revealed that the gap in different cognitive dimensions of different sexes gradually shrink with aging, which is consistent with the findings of a previous study, especially after 80 years of age, when men and women tend to decline at the same rate in various cognitive fields and no longer have significant differences [42].

Our research found that while men and women have different trajectory of normal aging in terms of different cognitive dimensions, these differences tend to diminish over time. This study had some limitations. This was an exploratory study with limited sample size and area, which cannot represent the changes in cognitive function of the entire elderly group aged 60 years and above in Guangzhou. Therefore, further large-scale studies are required to confirm the preliminary findings of this study.

## 5. Conclusions

Sex differences in cognitive variation indicates steeper decline for women on performance of verbal episodic memory, associative memory and short-term memory. Meanwhile sharper deterioration in memory, comprehension and recognition of spatial relationships, visual structure ability is observed in men. Discrepancies of sex differences in different cognitive dimensions diminished over time.

## Data Availability

This national multicenter study was conducted by the Shanghai Mental Health Center; therefore, we cannot share the database unless allowed by the organizers.
